# Misdirection and the Regulation of Herbalism in France and England

**DOI:** 10.23987/sts.110353

**Published:** 2022-05-15

**Authors:** Emilie Cloatre, Nayeli Urquiza-Haas

**Affiliations:** University of Kent, United Kingdom

**Keywords:** Herbalism, legalities, misdirections, science and law

## Abstract

In this paper, we propose to explore how the regulation of herbalism, in France and in England, rests on series of ‘misdirections,’ with the coexistence of law and herbalism depending on multiple magical illusions. Attempts to regulate herbalists, and the responses they invite, involve multiple sleights of hands both by the law and by herbalists. Herbalists perform misdirections to maintain an illusion of legality, even where they bend legal rules that they deem incompatible with their practice. But far from being the only, or even the most effective, tricksters, herbalists are only one set of performers in a more complex layering of legal illusions. The regulatory and legal infrastructure itself relies on misdirections enacted through everyday legal procedures that trick the general public into believing that the law is ‘acting’ to protect vulnerable consumers from dangerous healers and their medicines, while the effects of its actions may be to tolerate, or indeed produce, zones of illegal, or ‘barely legal,’ practices. At the same time, this performance is enabled by playing a disappearing act, in which the knowledge of herbalists, and the demands of their users, are disappeared behind the screen of apparent legal protection. Drawing attention away from competing claims to knowledge, and towards its protective intervention, the legal system thereby embeds misdirections of its own kind.

## Introduction

In this paper, we explore the regulation of herbalism, in France and England. We focus in particular on the tensions, mishaps, and frictions that the process creates, reading those as misdirections: crafted gestures that direct the gaze towards some action to make others less visible, enabling the disappearance of objects, practices and political claims. Since those who heal with plants have historically been considered as both powerful and threatening, herbalists have for long been seen as a profession in need of regulation. Yet, recent attempts to create new rules for herbalists, and the responses offered, involve multiple sleights of hands. On the one hand, the regulatory and legal infrastructure relies on misdirections enacted through everyday legal procedures that create a perception that the law is ‘acting’ to protect consumers from dangerous healers, even when it is not clear that this is its effect. On the other hand, some herbalists perform misdirections of their own in response to the rules meant to restrict or regulate their excesses, deploying minor disobedience as part of a toolbox of discreet activism. In the end, who is tricked and by whom is not easy to discern, and intentionality is itself difficult to capture: those who acknowledge that their own action involves some dissimulation see this as incidental to a different kind of meaningful process; others follow a quasi-ritualised procedure, where the disappearance is embedded into the politics of law itself. To explore those tensions, we approach the concept of misdirection as a heuristic to identify practices that may be construed as creating illusions of visibility and invisibility.

Exploring the difficult negotiations of herbalism with the law in France and England, we suggest that rules misfire in both countries, producing zones of illegality and of ‘barely legal’ practices, each with their particular characteristics. In France, the criminalization of herbal medicine practice by anyone other than a pharmacist has meant that herbalists always operate on the border of il/legality. When rules are bent (or more explicitly broken), it is not necessarily ill-intended, but an inevitable aspect of trying to work within restrictions that makes herbalist practice almost impossible, threatening its sustainability. In England, ambivalence towards herbalists’ knowledge has materialised into regulatory mechanisms which some factions considered to be mere bureaucratic ‘smoke and mirrors.’ Herbalists are tolerated and their practice survives within the narrow confines of a tightly defined legal exemption. And while most strive to comply with the law, and work within its boundaries or close to the edge, they are sceptical about its effects. The examples in this article explore the intricate rules that relate to the kind of products herbalists are allowed to sell, and how those products should be prepared, packaged or labelled; the advice herbalists can (or must) provide; requirements around place of sale and consultations and, to some extent, professional regulations. While some of these rules are very clear, others are less so, creating a wider zone of malleability and opportunities for negotiation, avoidance, or simulation. Whereby herbalists may be seen to more directly trick and distract, to offer a neat version of compliant action to legal agents who oversee their practice, the legal system also operates its own misdirections. Those are more layered, involving both displacement and the embedding of techniques of distraction, where intention and routinised performance are difficult to distinguish, yet herbalists’ particular epistemological and political claims disappear. The examples we use to illustrate these include the disconnect between the law on paper and the everyday tolerance of minor disorder; the invisibility of herbalists’ knowledge in debates around professional regulations and the sale of medicinal plants; and, in England, the regulatory illusions produced by the bureaucratic management of herbalists as a profession.

As we map these misdirections, we endeavour to show the possibilities, limitations and complexities of the interface between regulation and everyday healthcare practices at the crossroad of contested science and fragile legalities. The analysis builds on scholarship at the crossroad of STS and socio-legal studies, interrogating both the everyday practice of law in healthcare and the interface between law and scientific knowledge. It also engages work on law and magic, that has challenged the law’s ascribed identity as a pillar of rationality: in that respect, it echoes some of the concerns of law and STS scholars in interrogating the nature of knowledge in legal processes, while emphasising the ritualised processes that, in our case study, facilitate the disappearance of competing claims.

## Methodology

The forthcoming discussion is based on an analysis of the contemporary legal debates surrounding herbalism in France and England, both in formal legal settings and herbalists’ everyday practice. We rely on a mixture of legal and documentary analysis, and semi-structured interviews. Moments of increased political activity have generated significant documentary data, in the form of regulatory texts, policy reports, and parliamentary debates, initiated over slightly different periods in France and England. In England, these debates arose in the aftermath of a 2001 House of Lords Science and Technology Select Committee Report- which explored more broadly the regulation of Complementary and Alternative Medicines (CAM) in the UK- and concluded in 2014 after it was decided they were not ready for statutory self-regulation. In France, the debates arose in the context of two Senate Commissions- in 2012 and 2018- that re-opened former conversations about whether the State allow herbalists to professionalise and self-regulate, and how it should regulate the sale of medicinal plants. As well as policy documents, these events triggered numerous public reactions from herbalists via numerous blogs, online material and grey literature, and some media coverage, that we also reviewed.

Alongside written material, we rely on semi-structured interviews with 25 people, conducted between 2017 and 2019. Participants were selected for their involvement in relevant legal debates, or because of their oversight of the professional regulation of herbalists. They comprised of representatives of herbalists associations in England, and the main schools of herbalism in France (which, as well as their role in training and education, have been at the forefront of campaigning on behalf of the profession); representatives of regulatory agencies (in England) and parliamentarians (in France); and, through snowballing, individual herbalists who had been involved in relevant debates. Finally, this research is part of a broader project on the regulation of traditional and alternative medicine, that informs some of our analysis.

### Law, science and misdirections

Herbalism has been part of the healing landscape in France and England for centuries. Even as biomedicine has settled as the primary healthcare resource, medicinal plants continue to attract interest (Garreta, 1998). Herbalists thrive to be seen as the main experts of medicinal plants, but their place is unsettled: in France, they have no formal recognition as a profession, and much of their activities are seen as intruding on the spaces that are legally reserved to pharmacists (or doctors) (Campion, 2003; Bost, 2015). They straddle the boundary between legality and illegality, remaining within the former only as long as they abandon some of the core constituents of herbalist traditions (Adams, 2002; Cloatre et al., 2021). In England, the position of herbalists vis-à-vis the law is less fragile, and they benefit from a zone of tolerance via exemptions to medicines laws. At the same time, efforts to be formally regulated (and thereby recognised) as a healthcare profession has failed, denying in the process some of the particularities of herbalists expertise. As they continue to negotiate their place within the legal system, contemporary herbalists also defend a particular model of healthcare and a particular socio-political vision in which narratives of nature are layered over matters of health (Elzière, 1986; Garreta, 2007; Grisoni, 2012). Looking more closely at the relationship between herbalists and their regulation reveals a series of misdirections, sleights of hand that disguise or displace claims, actions and politics.

These movements are shaped by the social and epistemological particularities of herbalism, while echoing the broader, complex and sometimes uneasy, relationship between CAM, state institutions and biomedicine that others have pointed to (Adams et al., 2005; Brosnan, 2015, 2017; Vuolanto, 2015, 2018; Wahlberg, 2015). Both in France and England, contemporary herbalism has negotiated its identity with and beyond science: notwith-standing some diversity in individual positioning, herbalists have engaged with scientific knowledge and practices to demonstrate the technical validity of their practice, while retaining a certain attachment to tradition and nature in their discourses (Bost, 2015). Like other CAM professions, they have also sought to define a cohesive professional identity, even if such effort has at times been hampered by the diversity of their practices and epistemological standpoint (Brosnan 2017; Stöckelová and Trnka, 2020). Despite these efforts, public discourse has sometimes continued to reduce herbalism to a more straightforward form of popular practice, based on less rational beliefs than science. For example, a French Senatorial report on complementary therapies stated clearly that to be considered ‘medical’ in the eyes of the law, these therapies should become “a haven of rationality from which magical beliefs should be banished” (French Senate, 2013: 7). Most contemporary herbalist associations would reject the characterisation of herbalism as an extension of magic, a label that responded to another historical period when the use of herbs by female sages had been considered the work of the devil (Manderson, 2005). Instead, herbalist associations in England and France today emphasise their likeness to pharmacological sciences and to health practitioners who follow diligently a professional code of conduct (Banerjee, 2009; Evans, 2008; Vanmarie, 2002; Wadell, 2019). They often make the case that herbalism has a scientific basis, but one that has yet not been demonstrated through the exigencies of the medical and pharmaceutical industry and regulatory apparatus (Dreher, 1983). By adopting the codes of science and the medical profession, herbalist practice has sought to cement its legitimacy, including vis-à-vis legal institutions (Wahlberg, 2008). On this basis, in England and France, professional associations have endeavoured to convince ministers, legislative bodies and medical authorities that herbalism has sufficient scientific credentials to be regulated as such. At the same time, many herbalists, in their individual capacity, insist that herbalism is not purely reducible to science, emphasising instead its harnessing of the powers of nature, and a different kind of ethos of care, a message also echoed by some of the schools or associations when describing their approach (Bitcon et al., 2016; Scottish School of Herbal Medicine, 2021; Wadell, 2019). In this way, contemporary herbalism overlaps with scientific narratives, while also proposing an alternative vision of the interface between nature and healing, and between plants and humans, that is not reducible to scientific rationalities. How herbalists negotiate this duality is a matter of shared as well as individual practice: associations and individual herbalists adopt nuanced stances, from outspoken positioning within scientific discourse, to some emphasising their difference, echoing imaginaries of tradition, or, in some cases, of magic itself. Responses from policy actors similarly locate herbalism in different discursive registers, within or away from science.

This ambivalence of herbalism – perceived or enacted – partly explains the frictions that can exist between herbalism and law. Arguably, modern law has been built on a powerful assumption that it was first and foremost an exercise in rationality and foreseeability, and one of the pillars of contemporary states keen to shake off any remnant of their premodern, less rational selves (Pękala and Stępień, 2012). Laws and norms are assumed to be guided by objective, unemotional and provable knowledge and similarly applied coldly and systematically: their legitimacy is based on this understanding that they are objectively informed, transparent, and built on provable knowledge (Conaghan, 2013; Norrie, 2013). Legal norms are presented as having shed their magicoreligious origins and instead, ‘modern’ law is tied to the rule of rational democratic governments (Ziolkowski, 2003). But this has also meant that the law struggles to engage with practices that are not perceived as rational in this way. The difficulties for contemporary states to regulate witch-craft is maybe the most striking example of such difficulties (Geschiere, 2019; Petrus, 2010; Pharr, 1932; Roberts, 1935), but because of its ambivalent positioning, herbalism has posed a more discreet, yet partly comparable, set of frictions.

In its effort to position itself as a pillar of rationality, the legal system heavily relies on scientific knowledge and evidence. Legal authorities engage with numerous forms of scientific expertise to provide reasoned decisions, that are presented as the logical outcome of factual constraints, rather than the outcome of a political process (Jasanoff, 1990, 1995). Yet, in the way that STS scholars have approached science, and the relationship between sciences and complementary medicines, as the effect of a socio-political processes (Brosnan et al., 2018) loaded with implications and embedded patterns of exclusion and imbalance, others have demonstrated the politics of the relationship between law and scientific knowledge (Cole and Bertenthal, 2017). Rather than the interactions between law and science being a matter of transposing knowledge from one institution to the other, in order to foster rational decision-making, law and science can be seen as constantly shaping each other as institutions, anchoring their respective power over social relations while rendering less visible their individual politics, contradictions and limitations (Cloatre and Pickersgill, 2020; Jasanoff, 2006). In this process, the relative legitimacy of different forms of knowledge and sets of practices is constantly renegotiated, with those deemed illegitimate pushed to the side of the legal system while others are more comfortably fitted (Cloatre and Pickersgill, 2020). In the analysis below, we explore an example of such exclusion through the metaphor of misdirection: a trick of disappearance by maintaining the gaze of the audience elsewhere, hiding politics as well as a process.

As explored by other articles in this issue, misdirections are in some ways magical. However, law and legal institutions more generally have a complicated relationship to reason and ‘magic’. Legal scholars pointed out that the law is itself an institution rests on its own set of rituals (Goodrich, 1996). Exploring law’s own languages and codes, often not far from incantations, or the spectacular and ritualistic nature of trials, critiques have insisted that the legal world is riddled with quasimagical features (Barshack, 2000; Clark, 1930; Corcos, 2001). Indeed, and without denying its distinctive characteristics, approaching law as one form of ritualistic ordering alongside magic might be a more suitable way of thinking about its power to act, or to enchant, even those it fails to convince about its rationality. Insofar as the act of enchanting relies on ‘dazzling’ a spectator, in this reading, the rule of law does not necessarily derive from it being ‘rational,’ but in its ability to divert from features that may remind us of its more mystical foundations, and force our gaze on its ‘reasonable’ and ‘predictable’ nature: echoing Bruno Latour, the law has never been modern as much as it has worked to define itself as such (Latour, 2012). Law’s magical features largely operate because modern legal practices are a “secularized way of performing certain material effects through symbolical acts; rituals that work in the imaginary but have unmistakable consequences in the ‘real’ world” (Alvarez-Nakagawa, 2017: 1250). The ritualised magic of law adopts a particular form in what some have termed the post-regulatory state (Collin, 2004; Fletcher et al., 2019; Fries, 2008). A feature of contemporary regulation is that the governance of conduct is no longer controlled exclusively by centralised state institutions. Instead, legislative functions have become fragmented and dispersed across multiple institutions and social actors. Rather than ‘command and control’ directly the behaviour of the regulated, the state manages behaviours ‘at a distance’ by delegating some of its functions to institutions other than courts and parliaments (Black, 2002). The law is then not only found in legislative acts of parliament but instead, is dispersed in guidelines, codes of conduct, manuals, etc. This fragmented landscape of regulation, facilitates legal misdirections, a play of ‘smoke curtains’ and ‘mirrors’ where the law hides particular realities from view, away from the visible and spectacular theatre of politics, into more discreet and routinised spaces of legal decision-making that shape social experiences (Ball, 1975; Keenan, 2017; Rogers, 2008; Simpson, 1985). In our case studies, the law performs small tedious ‘abracadabras’ that dilute aspects of herbalists’ knowledge under the pretext of managing and preventing risks associated with the practice of herbalism, albeit in different ways in each case study. This is the case for example when layers of bureaucracy create an illusion of substantive regulation and epistemic ordering. In doing so, the legal system misdirects our gaze away from the socio-political stakes of the ordering of healing, towards a tidy narrative of order linked to a set of rational procedures of risk management.

Alongside the misdirections embedded into the legal system, we explore the more visible forms of misdirections that herbalists perform in their efforts to act in ways compatible with the law. Using the malleability of the law, they stretch its boundaries when they consider it necessary to provide products, advice or care that they deem essential to their practice. In our reading, these become part of a broader attempt to be visible and recognised, to challenge the tacit exclusion performed by the legal system. We explore what it means for herbalists to sit at the edge of the law (in the case of France) or to see some of their practices hampered by increasingly complex regulatory demands (in England). This position creates everyday frictions between the possibility of practising herbalism on herbalists’ own terms and remaining neatly within the boundaries of the law. The friction points also misfire into divergent trajectories that both enable, exclude and particularize different practices (Tsing, 2005: 6). The trickeries at play are facilitated by the state of regulation: in both contexts, the grey areas left by regulations, and the exclusions they perform inevitably place much everyday practice on the edge of il/legality. In this way, illegality can be seen as constitutive of legal logics, an expected part of the project of making law. This part of our analysis builds on socio-legal engagement with legality and illegality, and in particular, on the tradition of legal consciousness: the everyday practices of law, particularly how its users diverge, adapt, challenge, or adopt law creatively, are studied as part of the law itself rather than as an excess that could be reduced by tinkering with the law. Law is seen as relational and therefore bi-directional, inevitably being changed by those who engage with it, while they also experience alterations as a result of their direct and indirect encounters with the law. The key analytic shift, where the emphasis is not on law but on legalities, is understood by Ewick and Silbey as a focus on “sources of authority, and cultural practices that are commonly recognized as legal, regardless of who employs them or for what ends” (Silbey and Ewick, 1998: 22). This dissolution of law with a big ‘L’ is altered by the exploration of law in society, including how different social actors ascribe different meanings to what they consider legal or illegal, how they experience, play and redraw those boundaries, and ultimately, rewrite them (Cowan, 2004; Halliday, 2019). When we speak about the misdirections of herbalists, we intend to move beyond simple dichotomies on what is legal or illegal, lawful and criminal, and instead, understand how the misdirections of herbalists co-create spaces of juridical tolerance. These misdirections are a form of tacit activism that sustain world-making projects (Fritsvold, 2009; Halliday and Morgan, 2013). Building onto the insights of socio-legal and anthropological studies on illegalities, we assume there are supplementary ‘meanings’ of licit/illicitness construed by social actors, where the crossing of legal boundaries matters more in social than normative ways. Negotiating with il/legality may be interpreted as a survival strategy (Peterson, 2014), or as an alternative lay interpretation of the law that supports a different kind of ethical project where the law is seen as having failed (Cloatre and Enright, 2017). The act of law-breaking can also be part of more explicit activist projects, drawing attention to alternative lifestyles and alternative futures to those proposed by states and enabled by the law (Fritsvold, 2009).

In the following sections, we explain first the laws regulating herbalism in France and the main misdirections that we have identified. This is followed by a similar overview about herbalism in England, presented as a counterpoint to the French case. In each case study, we concentrate our analysis first on the misdirections herbalists perform to sustain their everyday practice and second, in the misdirections performed by the law through its regulatory bureaucracy and its disappearance of herbalism(s).

## Herbalism and the law in France

In France, products that are considered ‘medicinal, including medicinal plants and manufactured herbal medicines, can only be sold by pharmacists, and in pharmacies according to article Art. 4211-1,5, of the *Code de la Santé Publique*. Anyone else selling medicinal plants can be found guilty of the illegal practice of pharmacy, which is punishable under criminal law. Since the 1980s, herbalists have organised to contest this de facto monopoly, claiming that they too should have a legitimate role to play in the distribution of herbal medicines (Bost, 2015).

In response, and in the light of increasing demand from consumers, some exceptions to the pharmacists’ monopoly have been created over the years, in particular for plants thought to be innocuous. A 2008 law liberalised 148 plants from the pharmacopoeia and made them available for general sale (Journal Official, 2008). These can be sold in places other than pharmacies – often health stores, or one of the few traditional *herboristeries* that still exist. But there are restrictions on how those plants can be sold. For example, plants cannot be mixed (with a few exceptions of specifically authorised mixtures) and should be sold in raw form (except for a few that can be sold as powders). Importantly, only pharmacists can advise on how those products should be used: despite this concession made to those who wanted to sell plants outside pharmacies, pharmacists continue to be the only actors recognised to have expert knowledge over medicinal plants. Herbalists are allowed to sell innocuous medicinal plants, but only as can everyone else: the law gives them no additional right to advise or prepare. Effectively, their knowledge is not considered ‘special’ in any way, diluting their claims for recognition as legitimate experts of plants, as we return to.

But herbalism has not always been as constrained in France as it is today. Until 1941, certified herbalists occupied a legitimate (if fragile) place (Bost, 2018). After many years of pressure from the *Ordre des Pharmaciens*, the certificate was rescinded in 1941, which erased herbalists from any regulation relevant to healthcare practice. The fact that this took place under the Vichy government, though partly incidental as the reform was in the making for decades before, has come to sustain claims by herbalists that it should be seen as a historical anomaly that needs reversing. Consequently, from the early 1980s, a new generation of herbalists organised to try to have the certificate reinstated, with occasional support from politicians, but so far unsuccessfully (Cloatre et al., 2021). Today, herbalism is a coherent profession, unified in its effort to seek some recognition from the state, and to reclaim a more legal space.

### Tricksters and boundary-crossers

Herbalists have learnt to work within the relative invisibility conferred by the law while stretching the boundaries of what they are allowed to do. How the law defines who should sell medicinal plants maps uneasily onto their actual availability, and the practice of herbalism. Despite apparently clear legal boundaries, the trade of medicinal plants in France is messy, and much of it happens outside of pharmacies. Plants sold on markets, in health stores or specialised herboristeries, constitute part of everyday healing for many users (Garreta, 2007; Brousse, 2018). Although many of these plants belong to the list of plants authorised for general sale, not all of them do. Similarly, even though only pharmacists are, in theory, allowed to advise on the medicinal uses of plants, others provide forms of guidance that isn’t dissimilar. To some extent, this is because some herbalists respond to the constraints of law by playing tricks with the legal order, bending its borders in response to their needs, or the demands of users. Yet, rather than being about deception, these tricks are a form of negotiation and adjustment to precarious conditions, inevitable trade-offs to sustain traditions that, they fear, would otherwise disappear, or minor diversions from the letter of the law to deploy other registers of care or safety. If herbalists are not fundamentally animated by a desire to trick the law (and indeed have been engaging with state authorities to renegotiate the law), they are also concerned that current regulations are unnecessarily restrictive and counterproductive, and that their strict application would result in a distorted and unworkable practice of herbalism.

Even where herbalists cross legal boundaries, they tend to do so with measure, remaining within the law, or arguably within the law, as far as possible. They are aware of the law, particularly when they have been provided with formal training, readily available since the 1980s (Ecole des Plantes de Paris, 2021; Ecoles Lyonnaise de Plantes Médicinales, 2021). The boundaries of legality continue to matter, but may be bent when it is required to keep herbalism possible and meaningful. Boundary-crossing is also usually discreet, keeping a façade of legality even when its substance is debatable, behind which less legal endeavours may also take place. For example, herbalists are guided by the law in terms of which plants can legally be sold outside pharmacies: yet, some feel that they need to occasionally venture beyond these restrictions when the list stops making sense.^1^ They do so with caution, and discreetly, for example hiding some of those controversial plants in a dedicated cupboard only to be opened for trusted customers.^2^ Authorised plants are displayed more prominently than the non-authorised ones, making it less likely that they might be noticed by anyone carrying the type of light touch checks that, in practice, often constitute the only way to be ‘caught.’ Or some might encourage patients to grow in their own garden plants that they are not allowed to sell, respecting the letter of the law while deploying a different understanding of the riskiness of plants (e.g. see the blog: D’Herboriste, 2019). Their motivation is rooted in the feeling that the law is poorly designed, creating a threat to herbalist practice through its blind spots. They consider the list to be ill-adapted and inconsistent, leaving out many of the most commonly used plants in traditional herbalism, or featuring only the less useful parts of specific plants.^3^

Similarly, their everyday work is constrained by the limits to the sort of claims they can make over healing and the advice they can give, since providing a diagnostic or health advice could bring a claim that they are acting illegally by undertaking acts legally reserved for pharmacists or doctors. Yet, selling plants without guidance or advice is seen by herbalists as problematic, a blindspot of the law that may expose users to risks rather than protect them.^4^ Again, many herbalists comply with this requirement. Others apply some flexibility, seeking to remain within a zone of tolerance while providing expert guidance. They may play with words to define such guidance so that it is not construed as ‘medical/medicinal.’^5^ Any guidance provided through labels is similarly cautiously phrased, avoiding medical claims or explicit posology.^6^ Such crafting can blur the boundary between health advice of the type reserved for pharmacists and doctors, and a broader type of non-expert suggestions provided to customers, and the boundary of il/legality.

In all these techniques, the aim is to direct attention away from practices that signal any boundary crossing: language, writing, objects are adjusted and moved around to suggest that nothing of (legal) significance is worth noting. Those who tease the boundaries of the law try to avoid attention (of pharmacists who may report them, of law enforcement officials) by playing subtle visual and spatial trickeries, creating a sense of doubt about what is at stake. But herbalists are also not the only actors enabling this pushing of legal boundaries to take place. If we are to seek intentionality in this particular misdirection, deviations are fostered and facilitated by the make-up of the law itself: the absence of statutory regulation and the lack of legal existence of herbalists in France means that much of their practice takes place in less regulated spaces. It is easy for borderline activities to go unnoticed because performances tend to be to a limited and sympathetic audience. Yet, these activities are not invisible: state agents or professional associations occasionally intervene, and stories of those who got caught and faced legal consequences travel far and fast. But such interventions are the exception to a more fluid everyday where rule-teasing is a secret hidden in plain sight: in that respect too, it resembles more a case of negotiation than of deception in ways that others have pointed out in their own analysis of il/legality (Cloatre and Enright, 2017; Cooper, 1996). For the most part, this negotiation does not prevent the broader infrastructure in which herbalism operates from holding up: in the day to day, most negotiations with the law and routine misdirections result in relatively peaceful and harmless coexistence between herbalists that monitor their own boundaries and legal agents that provide them with a zone of tolerance.

In this context, the stakes of the occasional boundary-crossing performed by some herbalists, and of this ongoing negotiation, can also be understood as part of a broader project of legitimation and resistance (Cooper, 1996; Fritsvold, 2009; Halliday and Morgan, 2013). Rather than being read as meaningless law-breaking, it is closer to a form of tacit activism, that seeks to expose and challenge the impact of the law on the ability for herbalism to survive (Cloatre and Enright, 2017). The ongoing efforts deployed by herbalists to renegotiate their position in law have been hampered by their ongoing precarity: attempts to be visible, and efforts to relaunch the profession, are made more difficult by the very strict limitations placed by the law on what they can do. In response, various individual and collective tactics have been put into place (Certeau, 2013). Stretching the boundaries of the law belongs to the former, with individual herbalists adopting different positions, some adhering to the strict boundaries of the law while others occasionally cross them, to sustain their ability to practice, and the future of their professions, and to remain visible and relevant. In that respect too, herbalists boundary-crossing is not mainly about breaking the law: rather they work through its blindspots, confident that their knowledge of plants means that they can safely circumvent the law while helping their patients/customers. They see patients as the main losers in a system that is so restrictive in terms of access to plants and plant-based medicines that they are more likely to be tempted to purchase treatment in less safe spaces, such as the internet.^7^ Their bending of legal requirements is a response to these limitations of the system, a way to overcome what they see as unfair and harmful effects of a misadjusted legal system.

### Disappearing knowledges as regulatory misdirection

Alongside their occasional performance of minute legal misdirections, herbalists have developed more collective strategies to renegotiate the law, bringing its incoherence to the eyes of the state through official routes, from the lobbying of individual officials to contributions to public conversations. This has been supported by careful strategies to redefine the common ground of their profession, with schools of herbalism developing extensive training for those seeking to join the profession, formalising the kind of knowledge that contemporary herbalism rests upon (Cloatre et al., 2021). So far, these efforts have been thwarted by a different set of tricks played by the legal system itself. Rather than the tricks, illusion and invisibility at play being the work of an identifiable trickster, however, those have a more systemic origin, embedded in modes of action of the law itself.

Despite the best efforts of herbalists to be given a space in the law, they have so far met limited success. Some individual MPs have been receptive to their demands, raising their concerns in parliament through parliamentary questions or, more significantly, two dedicated Senate commissions. But herbalists struggle to see their knowledge recognised as a particular form of expertise, and the minor bending of the law by some herbalists coexists with a more structural process of disappearance, where the politics of law become hidden under a cloak of science. The legal system operates on a series of misdirections, turning attention away from some matters, to direct it towards its seeming intervention or its claims to action, and away from its politics and tacit exclusions through an emphasis on scientific resources. One of the most effective ‘tricks’ of the legal system is to divert away from the particular type of knowledge that herbalists claim to possess, and their users wish to rely on. Through rhetorical and procedural manoeuvres, proposals by herbalists and users that a different kind of healthcare might exist, and that it might rest on a particular kind of expert knowledge, are discreetly effaced, disappearing behind the more forceful presence of scientific and biomedical demands.

This is in part because herbalists’ knowledge is a challenge to regulators in France: regulators and the politically influential medical and pharmaceutical councils regard it as being popular rather than scientific knowledge. As a result, it continues to sit uneasily with the scientific expectations on which the regulation of medicines and healthcare professions is otherwise organised, and indeed the type of rationality on which modern law tends to rely. This tension, already ingrained in the laws that effectively consider herbalists as no more knowledgeable about medicinal plants than anyone else, has also hampered herbalists’ efforts to be regulated otherwise: the boundaries of legitimate knowledge proposed by the law do not align with those followed by herbalists nor their customers. This disconnection has been striking whenever the question of herbalism has featured in parliamentary discussions. Since the 1980s, the (re)creation of a herbalist certificate has been occasionally raised through parliamentary questions. Each time, the response provided is the same – a cut and paste answer that brushes aside the possibility of a substantive discussion by rendering its problematique irrelevant: the certificate was rescinded in 1941, and medicinal plants are now sold only by pharmacists. In the eyes of the state, pharmacists have “complete knowledge of medicinal plants, in relation to their composition, pharmacological effects, and therapeutic uses” (Assemblée Nationale, 2020). This position negates the claims of herbalists or their supporters for a different kind of knowledge, focusing instead on the ‘complete knowledge’ that pharmacists possess. This was fleshed out further in the context of senatorial commissions, where (at the initiative of Senator Joël Labbé) the question of the diploma- and the future of herbalism in France- was explored in more detail. The commission proceeded with extensive interviews with a broad range of actors – including public agencies, Medical and Pharmaceutical Councils, industry, and schools of herbalisms – juxtaposing the claims and positions of different interest groups, and illustrating the coexistence of different visions for medicinal plants. In these conversations, those who opposed the re-creation of a regulated profession for herbalists (notably representatives of the medical and pharmaceutical Councils)insisted that there was no need for such profession because pharmacists already fulfil that function. Pharmacists were the ‘true’ experts of medicinal plants, fulfilling any possible need for herbal medicine (e.g. French Senate, 2018). In these exchanges, like in the standard response Ministers have offered to parliamentary questions on herbalism, what becomes evident is that attention to the knowledge that pharmacists possess also renders invisible the alternative types of knowledge that herbalists want to see valued. Herbalists do not claim to know about plants in the same way as pharmacists do; their claims are underlined by a different kind of health politics, also made irrelevant by insisting on the ability of pharmacists to respond to all needs. These claims do not deny the value of science nor scientific knowledge, nor its lack of relevance to herbalism itself: indeed over the years, herbalists have made significant efforts to situate their own knowledge within scientific paradigms familiar to the legal system. Schools of herbalism reach out to science by introducing relevant teachings into curriculums, working with suppliers, manufacturers and producers who align with pharmaceutical regulations and learning from science where they see it as complementary to their practice (Bost, 2018). They seek to adhere to epistemologies that can make them more visible and more acceptable to the codes of the law. But they do so without entirely abandoning their attachment to the less explicable powers of nature or the roots of their practices in popular traditions: they consider those to also be relevant to how we relate to and engage with plants, and how we can preserve more fully their powers to heal, in their many dimensions. This includes a certain scepticism towards how plants are envisaged and transformed through pharmaceutical processes, and a wariness of the industrial logics that underpin pharmacological uses of plants. It is in this respect that their vision for a different kind of herbal healing is political, and lost in an emphasis on the knowledge of pharmacists over plants as being ‘complete.’ This disappearance is effected by the legal system, not through direct confrontation, but through the repetition of what is seen as a straightforward, apolitical fact: expertise over plants is already supported by the law, and this expertise is all that patients may need. This is not to say that individual agents are seeking to deceive, or that they are themselves always actors rather than the audience in the legal misdirections at play: one of the strengths of legal misdirections is to rely on grander narratives and performances, within which everyday actions may individually be as expected, yet their association generate exclusion and fragility.

The effects of this disappearing act are read differently by herbalists and the legal system. For legal actors, it is a necessary step to protect patients from the dangers of plants. But by refusing to reach into the world of those who are seeking from herbalism something explicitly different from what pharmacists have to offer, and side-stepping suggestions that knowledge over plants may be multiple, the legal system also triggers some exclusions. Its denial of alterity leaves users dissatisfied with what pharmacists can provide needing to turn to less visible spaces on the edge of legality, where their only protection is the type of self-regulation herbalists have sought to develop. For herbalists, this is the most problematic side-effect of the regulatory system: although it ardently portrays itself as designed to protect vulnerable users, its apparent strictness distracts from its own limitation. Here, as in other areas of law, vulnerability is turned on its head: whereas the law’s explicit aim is to protect, its ill-adjustment to everyday practice can result in fostering yet further risk and vulnerability, including by enabling zones of illegality (Munro and Scoular, 2012).

## Herbalism in England: misdirections and routinised bureaucracy

In England, herbalists have benefited from a common-law exemption to make herbal medicines, which means that they are not exposed to criminal law in the way French herbalists are (MacLennan and Pendry, 2011). Because of this, England was seen by the French herbalists we met as a more welcoming, as an idealised regulatory landscape.^8^ The additional space given to herbal practice means that herbalists in England don’t face the same threats of illegality, and the precarity they experience is of a different kind. They operate within a narrow space of practice allowed by a strictly defined legal exemption. This makes legal misdirections less striking, even more openly akin to a negotiation of boundaries. Areas of opaqueness proliferate along the margins between acceptable and less acceptable practices, which some herbalists feel the need to stretch to sustain what they see as meaningful practice. And for all its apparent efforts to recognise the kind of social demands that herbalism addresses, and put in place a detailed apparatus to protect those who choose to rely on it, it is not clear that the regulatory system has been willing to engage with herbalists knowledge, or their claims for difference, much more than the French system has.

Today, under the herbalists’ exemption, subject to certain conditions, anyone can prepare, give, and sell herbal medicines (based on single or multiple herbal substances or preparations) in the context of a one-to-one consultation.^9^ Some limitations apply: the herbal product should not be manufactured or assembled by a third party; the supply of the herbal remedy ought to be done in the same premise where it was assembled, and if using restricted herbs (for example, aconite, chinchona bark, ephedra), those must be kept safely away from the public (Medicines and Healthcare Regulatory Agency, 2014). Importantly, what herbalists can do can also legally be done by anyone else, because the herbalist title is not protected by law (Banerjee, 2009; Clarke et al., 2004).

The restrictions under which herbalists in England need to operate foster their own type of boundary-work that stretches the law in discreet ways, bending the borders of legality while appearing to be in full compliance. This is illustrative of the kind of negotiation fostered by lay engagements with the law. For example, changes to the legislation in 2012 brought by EU rules prevented herbalists from acquiring bespoke preparations ordered from third parties, causing practical difficulties to herbalists who had been relying on such supplies (McIntyre, 2011; Santosh, 2015) before the gradual shift away from common law.^10^ One herbalist we interviewed recalled researching the wording of the law, trying to figure out a way to stretch the boundaries of the meaning of ‘manufactured’ herbal products:

For a while you see under the 1968 Medicine’s Act, I was trying to find a way that we could continue to legally practice. And there was a part in the Medicine’s Act where I think trawling through the Medicine’s Act sort of midnight one night and I came across the words about assembly and the words assembly were in that Act which said, as long as the product is assembled on the premises. And I thought, well I wonder what the actual legal term ‘assembly’ really means. I sort of trawled down, trawled down and sifted through the whole thing until my eyes were popping out and found that ‘assembly’ according to that definition meant putting a label on it, which seems ridiculous, but that is what it came down to. So I thought, well if we can get our external herbal suppliers to make up the prescription and send it back to us, we stick the label on and give it to the patient. We are still working within the law because the final assembly is taking place on our premises […].^11^

Although she did not actualize this potential misdirection, this shows how herbalists can construe their practice through the finer components of legislation, looking for ways to expand definitions to make essential elements of their practice sustainable. Law is also produced and transformed through this craft: the letter of the law might be the purview of judges and regulators, but social actors find alternative meanings and construct legality out of continuous evolution in their relationship with the law (or in the absence of it) (Halliday, 2019; Hertogh, 2004). Such interpretations can go untested and unchallenged until the more exceptional intervention of formal legal actors, but in the everyday of the law, such interventions are not the norm. Another participant talked about how her health store offers light-touch ‘consultations’ at the till, rather than in the private settings that the law requires.^12^ This practice has been found in local studies in London too among Chinese herbalists (Teng et al., 2015). This was a way for her to work around restrictions to continue selling mixed herbal remedies, a key aspect of her practice. When explaining such negotiations, herbalists were conscious that they are somehow stretching the rules. Yet, like their French counterparts, they are also keen to try to avoid direct confrontations with legal institutions, looking for ways to make the requirements of the law workable for their everyday practice, and when this is not possible retaining as much of the spirit of the law in their adjustments as they can. Like in France, these minor misdirections are enabled by the regulatory system, and its fostering of zones of tolerance: most infringements carry little sanctions, and legal cases against herbalists have been rare. Rather than acts of law-breaking, the stretching of legal boundaries is a performance in which actors and audience play their part, as long as a degree of care and measure remains applied.

### Disappearing knowledges as regulatory misdirection

In very much the same way as their French counterparts, herbalists in England strive to show the relevance and particularity of their knowledge and to demonstrate it apprehends some aspects of healing differently from biomedical professions. If they accept that there are some overlaps in how different constituencies may know about plants and their healing power, they also revendicate a unique contribution and advocate for the survival of these modalities of healing. Yet, the regulatory system disappears herbalists knowledge as expertise, in ways that are more nuanced but not dissimilar to the French context.

In the last twenty years, herbalists’ attempts to become a profession regulated by statute have failed. To some extent, their goal got caught into a broader deregulation agenda pursued by the British state, aimed at limiting statutory instruments because they were considered expensive, bureaucratic and ineffective (Allsop and Jones, 2018; Hampton, 2005). But by applying this logic to herbalism, the state performed a misdirection of its own: it evaded and postponed any serious engagement with claims of expertise by those who use plants as medicines. Legislators and other stakeholders in the regulatory debate have drawn attention to herbalisms’ absence of a scientific basis or that those who make herbal medicines don’t have a standardised and homogenous body of knowledge that transcends cultural differences between healing traditions. The lack of homogeneity among those who use medicinal plants continues to sit uneasily with the universalist scientific expectations on which the regulation of medicines and healthcare professions is otherwise organised, and indeed the type of rationality on which modern law tends to rely. For example, the 2001 House of Lords Science and Technology Committee report segmented professions into legalisable and non-legalisable professions (Banerjee, 2009; House of Lords Select Committee on Science and Technology, 2000). Here, likeness to science- its ontological, epistemological and operative standards- became a way to discreetly sideline claims by non-European medical traditions. Western herbalism was considered more legalisable because of its closeness to scientific language and epistemological basis (Cant, 2020). By contrast, practitioners of Ayurveda and Traditional Chinese Medicine were deemed non-legalisable professions, and even described as potentially ‘dangerous,’ because their knowledge was more akin to philosophy or religion. In the end, any hopes herbalists may have had for statutory regulation had to be abandoned.

It could be argued that herbalists’ scientific character has been shaped by their exclusion from the healthcare system. Critics have long redirected attention towards the ‘unscientific’ character of herbalist practices to justify its regulation or prohibition. And in response, herbalists have tried to become more like doctors or like pharmacists: they have set up associations that set standards of conduct, they have incorporated scientific norms and practices in their education and training and they have promoted the integration of research about the therapeutic effectiveness of plants. Some of these associations have lobbied successive governments over the last hundred years to have their title protected. Protection of title serves two key objectives: it prevents non-experts from practising and unsafe practitioners can be banned from a register. Yet, herbalists have nevertheless failed in their attempts to convince parliament of the need to protect their title. This might seem surprising, considering herbalists have been somewhat tolerated and they have had allies in parliament who have been sympathetic to their plight. However, tolerance has not been born completely out of trust in herbalism *per se*. Many times, it has been defended on other terms: for example, parliamentarians and other government institutions have defended herbalism on the basis of the freedom of choice of consumers and legislators own conception of Britain as a liberal country (e.g. House of Commons, 1985).

Despite herbalists attempts to have their profession protected by law, they have also rejected offers where their identity is at risk of being erased by becoming assimilated or subordinated to the medical profession. When Aneurin Bevan offered herbalists to join the National Healthcare System in 1948, herbalists rejected it because they would have had to fall under the oversight of doctors (MacLennan and Pendry, 2011). Today, herbalists and traditional healers using medicinal plants also have a plurality of voices and positions concerning such regulation. Most associations have been enthusiastic about regulation and embraced transformation into a more ‘scientific’ practice aligned with the law, and one that promotes the use of over-the-counter traditional herbal products when properly regulated. However, some factions have been more ambivalent about the benefits of becoming regulated by law, and have actively resisted the ‘scientization’ of herbalism because such a change would disappear the very knowledge and practices they have embodied (e.g. see the blog: Herbarium, 2009). Those involved in these networks prefer to stand at the edge of the law and sometimes stretch some of its rules. One of the herbalists even parodied the scientific identity she encountered in her university education by wearing laboratory coats and acting out at the same time ‘witch-like’ behaviours.^13^ This re-appropriation of the exclusion is also a resistance to the pull to become subjected by the symbols of scientific authority (Loizidou, 2007). That same herbalist also used the symbol of magic as a way of reembracing the otherness of herbalism and deflecting criticism from what is understood as a scientific standpoint:

When we first qualified and we first went out onto the market stall, we were met with a lot of people kind of saying, can you prove that it works? How do we know? We responded by bringing scientific research, our papers and they just even when you have got all of that, they still want to push and go, how do you know it works? What is it? And then, one day, I don’t exactly know how it came about, but we made witch’s costumes and we put black pointy hats on and we had a cauldron and then nobody ever asked us if it works anymore. All those people were just kept away.^14^

Regardless of herbalists’ chosen response, the regulatory system has two limitations: the first is the implication that herbalists knowledge needs transforming, along epistemological lines that do not necessarily sit well with some of their beliefs. In this process, claims to be ‘otherwise’ are discreetly silenced in favour of a conditional acceptance, that depends on embracing more scientific paradigms (Dixon, 2014; McIntyre, 2011). The second is that, even if herbalists are to embrace new regulatory demands, unless their title is protected anyone could claim to be a herbalist regardless of their own credentials. Herbalist knowledge remains at best tolerated but is not seen as sufficiently palpable to be protected as expertise. Yet, the complex bureaucratic apparatus that was created as an alternative to statutory regulation makes such tacit exclusion, and lack of protection, hardly visible. This is arguably the most striking type of misdirection undertaken by the regulatory system, and one that may be most common to contemporary societies: bureaucratic routines are deployed as a way to diffuse and disappear more complex socio-political claims, and epistemological debates.

### Bureaucracy and the deflection of debate

While herbalists had sought to be offered the legitimacy and protection conferred by professional statutory regulation, the British government provided a rather different response. In 2011, the government announced that it would create a register of practitioners using unlicensed herbal medicines, to be overseen by the Health and Care Professions Council (HCPC) (Barber, 2014). Herbalists and traditional healers thought they had finally succeeded in their efforts for state recognition as a scientific discipline. As one of our interviewees noted, the HCPC was “the natural home for herbal medicine, because it is setting standards. It is about making sure that people can’t practice if they are struck off.”^15^ However, this decision was overturned in 2014. Instead, herbalists were brought under the oversight of the accredited register program of the Professional Standards Authority (PSA) (Wadell, 2019; Walker, 2015), triggering a rather different, and more bureaucratic approach.

Set up in 2002, the PSA is an independent meta-regulator that ‘regulates the regulators.’ Meta-regulators imply re-casting the function of law from direct control to proceduralisation (Scott 2004; Aust and Gozlan, 2010). For example, in the case of the PSA, one of its functions is to oversee the processes for dealing with complaints and standards used by all health professions, including doctors, nurses midwives, etc., without directly engaging with the professions themselves (Allsop and Jones, 2018). Instead, it oversees the procedural systems that other regulators have put in place.^16^ This oversight by a dedicated agency enables the state to scale back traditional legal mechanisms, relying instead on less direct means of shaping professional behaviours through standards, guidelines, codes of conduct, education, etc. Self-regulating bodies- such as those created by the accredited registers scheme - have to follow a set of standards and processes to identify risks posed by registrants and mechanisms to mitigate them (PSA, 2015, 2018). If they fail to uphold such standards, the self-regulating bodies themselves will lose their accreditation, and, it is implied, their legitimacy. While the goal is to improve standards of practice, the day-to-day working has produced something rather different, where regulatory choreographies become an end in and of themselves. For some of the herbalists we interviewed and their associations, the focus on procedural actions risked missing out on substantive controls needed to protect end-users from unqualified healers, producing an illusion of regulation rather than any meaningful oversight. This approach to regulation assumes that the risks of unregulated professions are manageable through bureaucratic control focused on policing behavioural norms to manage the indeterminacy in healing relationships (Doyal, 1990; Gjengedal et al., 2013). But it does not engage with the substance or knowledges shaping the practices at stake, leaving aside the more difficult questions raised by herbalists’ demand for statutory regulation, since the knowledge(s) on which they rely is not relevant to the exercise. In its practice, this form of regulation may have made complementary medicines visible and knowable, and thus, “amenable to measurement, verification and validation,” (Wahlberg, 2015: 13) but it does little to engage their epistemological claims, nor to offer direct protection to practitioners or users.

This subtle misdirection sidelines politically thorny questions about herbal medicine’s efficacy and knowledge-base and redirects attention to the governance of standards, guidelines and other processes. On one hand, what unfolds is a more insidious and fragmented system of regulation that fails to engage with herbalism and other traditional healing knowledges on their own terms. Herbalists are not swooned by the charm. Most herbalist associations rejected this option, voicing their opposition to a system they deemed to be a mere regulatory illusion or a duplicate of what they were already doing (Dissenting Members of Herbal Practitioner and Medicines Working Group, 2015). It remains self-regulation without any ability to punish intruders and those who they deem potentially dangerous practitioners because the state has not protected their title. They regard it as regulation without any teeth, lacking any ability to correct risks associated with the use of plants, and side-stepping the main argument in favour of regulation: protecting the specialist knowledge required to mix and use plants correctly. At the same time, leaders of herbalist associations interviewed worry that embracing the PSA model would mislead people into believing that there is effective regulation.^17^ Today, two decades after the parliamentary inquiry that opened the door to the process towards statutory self-regulation, herbalists suffer from legislative fatigue and have a hard time believing in the magic of the law. They have not only figured out that the apparatus of professional governance is a magic trick, meant to create the illusion of accountability and enforcement of the rule of law, but they’ve also realized the magician is a trickster, a figure with no real power other than from those who believe in it and its rituals. Instead, they prefer to continue to practice away from its performance.

## Conclusion

Overall, both in France and England the interface between law and herbalism is riddled with misdirections: both regulators and herbalists foster a situation in which an illusion of cohabitation between legal logics and herbalist practice is provided. Yet, such cohabitation is resting on efforts to make others look elsewhere: herbalists negotiate their precarious activities between more or less visible registers, playing on the ambivalence of the law where they feel this is needed or justified. Regulators, willingly or not, disappear the particularities of herbalist knowledge and their users’ demands, denying some of their more political claims for epistemological alterity. In these debates as elsewhere, scientific knowledge and rationality deflect attention from other calls and voices. Herbalists, and patients who seek their advice and resources, are voicing dissatisfaction with what is available elsewhere, and indeed with biomedicine. Those who reaffirm the adequacy of existing regulation through a reminder of the range of biomedical resources available, or propose ways for herbalists to be more like (biomedical) health professionals, are drowning rather than engaging those dissident voices.

An effect of this situation, that herbalists most critical of the law we met decried, is to foster precarity and vulnerability, of herbalists as well as of patients. This in itself could be read as a form of misdirection, though unintended, inscribed in the logic of illegality itself: as claims and lifestyles are left out of legal debates, and pushed to the fringes of legality, they are displaced into less visible and less protective spaces. For herbalists, the biggest failure of the system is that no legal barriers are placed to prevent healers they consider less qualified, or less scrupulous, from practising. In pointing to this blindspot, they denounce some of the misdirections produced by law, hiding away the very possibility of ill-intended tricking that it fosters. The law claims to protect through restriction, criminalisation or selective legitimation, insisting on its completeness and the value of its rituals. Yet it leaves in its blindspots those whose world-making projects are deemed irrelevant or incompatible, relegating them to less legal or unregulated, and less protected, spaces. As the fragile status quo remains, herbalists and legal agents continue to perform everyday misdirections that, though multiple in their forms and mechanisms, open up questions about the nature of both herbalism and law. Each one plays into the game where both play expected roles, but in everyday life, some fall short of that ‘ideal’ and others take their role too seriously, to the point they believe their performances are real. Or one may say, with time, they become ‘real’ as reinterpretations and adaptations between herbalists and regulators become sedimented through practice (Butler, 1988; Callon, 2010). In the negotiations between herbalists and the law, broader questions of national politics also shape the kind of performances at play. In France, the background is in part about institutional tensions: changes to the rights of herbalists also touch on the sensitive question of where the exclusive rights of pharmacists and doctors extend, and where the monopoly of pharmacy over borderline products may be eroded. At the same time, the apparent attachment of the state to narratives of science, and of protecting through science and law, echoes broader expressions of Republican values. In England, bureaucratic rituals are part of a broader turn towards decentralised governance, where substantive decision-making can become dissolved in more mundane techniques of surveillance.

The performative coexistence of law and herbalism clashes with the portrayal of law as a pillar of rationality. Despite its continued reliance on scientific institutions as norm-productive, and on bureaucratic procedures as productive of a particular form of protection, ongoing negotiations expose the limits of law’s grander narrative as rational, predictable and transparent. A key feature of law’s magic is its ability to create fictions of separation and indivisibility where there is entanglement, yet everyday frictions expose some of the tricks at play. Insofar as the purification process is prone to fail or misfire (Callon, 2010; Latour, 2012), an everyday legal misdirection disrupts the gaze away from key political demands, while creating the illusion of ‘action.’ Driven by consumer desires, global herbal medicine markets thrive (Banerjee, 2004; Barnes et al., 2007; Kudlu and Nichter, 2019) and states are pressured to establish accountability mechanisms without willingly sanctioning herbalists knowledge. These tensions map onto narratives of the disenchantment with modernity, including how law participates and is one of its main adherents. The disenchantment operated by the law seeks to purify practices from the irrational, but in doing so fails to acknowledge the cohabitation of modern law with other ways of being. Weeding the magic out of herbalism, relocating it carefully into the remit of science, is an easier way for the law to tame it as an object and make it manageable. In turn, resistance to regulation is in a way a form of rebellion directed against the disenchantment of the plant world. Legal discourses take part in the stories of disenchantment characterised by the use of rationality as a replacement of magic, and predictability as a replacement of wonder (Bennett, 2001).

## Notes

1 Interviewee Fr.3 25/02/2018; Interviewee Fr.4 25/02/2018; Interviewee 1 06/04/2017.

2 Interviewee Fr.6, 10/04/2018.

3 Interviewee Fr.9 06/09/2018.

4 Interviewee Fr.8 06/09/2018; Interviewee 2 Fr. 06/04/2017.

5 Interviewee Fr.7 12/08/2018.

6 Interviewee Fr. 4 25/02/2018.

7 Interviewee Fr.6 10/04/2018; Interviewee Fr.8 06/09/2018.

8 Interviewee En. 5 05/07/2018.

9 Human Medicines Regulations 2012, Regulation 3 (6) and (9).

10 Interviewee En.2 29/05/2018; Interviewee En.3 05/06/2018; Interviewee En. 8 12/11/2018.

11 Interviewee En.8 12/11/2018.

12 Interviewee En.5 05/07/2018.

13 Interviewee En. 6 12/07/2018.

14 Interviewee En. 6 12.07.2018.

15 Interviewee En. 9 16/11/2018.

16 Interviewee En. 1 27/04/2018.

17 Interviewee En. 2 29/05/2018; Interviewee En. 8 12/11/2018; Interviewee En. 16/11/2018.
